# Oral Health Status and Dental Needs in Individuals with Sensory Impairments: A Systematic Review

**DOI:** 10.3390/jcm15155765

**Published:** 2026-07-23

**Authors:** Tara Kurpez, Ralph de Vries, Livia Naomi Faverey, Jelena Dumančić, Henk Simon Brand

**Affiliations:** 1 Centre for Special Care Dentistry, 1081 LA Amsterdam, The Netherlands; l.faverey@sbt.nl; 2 Department of Oral Biochemistry, Academic Centre for Dentistry Amsterdam (ACTA), 1081 LA Amsterdam, The Netherlands; h.brand@acta.nl; 3 Medical Library, Vrije Universiteit, 1081 HV Amsterdam, The Netherlands; r2.de.vries@vu.nl; 4 Department of Dental Anthropology, School of Dental Medicine, University of Zagreb, 10000 Zagreb, Croatia; dumancic@sfzg.unizg.hr; 5 Department of Dental Medicine, University Hospital Centre Zagreb, 10000 Zagreb, Croatia; 6 Department of Biochemistry, Faculty of Dentistry, Carol Davila University of Medicine and Pharmacy, 020021 Bucharest, Romania

**Keywords:** systematic review, oral health, dental needs, sensory impairments, hearing impairment, visual impairment

## Abstract

**Background**: Individuals with sensory impairments often encounter barriers to oral health information, preventive care, and dental services. Despite a growing body of research, oral health among individuals with hearing and visual impairments has not been comprehensively evaluated across the full spectrum of oral health outcomes. This systematic review aimed to evaluate oral health outcomes and dental treatment needs in individuals with hearing and visual impairments. **Methods**: A literature search was performed in Ovid MEDLINE, Ovid Embase, and Web of Science from database inception to 8 January 2026, in collaboration with a medical information specialist, and in accordance with the PRISMA 2020 guidelines. Observational studies reporting oral hygiene, periodontal status, dental caries, dental needs, and other relevant oral health outcomes in individuals with hearing and visual impairments were included. Given the substantial clinical and methodological heterogeneity across the included studies, findings were synthesized using a narrative approach. **Results**: A total of 5650 records were identified, of which 84 studies were included. Oral health problems were frequently reported among individuals with sensory impairments. Dental caries was the most common finding. Poor oral hygiene, gingival inflammation, periodontal problems, and unmet treatment needs were also often described. Additional findings included traumatic dental injuries, malocclusion, salivary and microbiological alterations, functional oral limitations, and reduced oral health-related quality of life. **Conclusions**: Individuals with sensory impairments are likely to experience poorer oral health and considerable unmet dental needs. These findings highlight the need for improved preventive strategies, effective communication approaches, and more inclusive dental care.

## 1. Introduction

Oral health is an integral component of overall health and well-being. Aside from being free of oral disease or pain, oral health can impact eating, speaking, and social interactions with others. Therefore, poor oral health can lead to negative physical, functional, and psychosocial sequelae if left untreated, which may be more prevalent among those who face barriers to healthcare and preventive services [1,2]. Oral health has been identified as a vital component of overall health and well-being in World Health Organization (WHO) reports [1,3]. Many individuals who live with disabilities experience health disparities including oral health disparities [2]. Disability as defined by the WHO encompasses impairments, activity limitations, and participation restrictions. In line with this definition, hearing impairment and visual impairment are considered sensory disabilities that alter the way individuals communicate with others, receive information, and interact with their environment [4]. Similar experiences may translate to oral healthcare. Hearing loss can create barriers to oral healthcare through verbal communication, and visual impairment can lead to decreased exposure to oral health information and prevention messages that promote daily oral hygiene behaviors [5,6]. Hearing and visual impairments can influence communication with dental providers, dental visit attendance, and preventive oral health practices [7,8]. As a result, hearing loss and visual impairments can place individuals at increased risk for poor oral health outcomes and higher dental treatment needs. Research has shown that individuals with hearing impairments and visual impairments have poorer oral hygiene, increased rates of dental caries, and greater unmet treatment needs when compared to their peers without impairments [5,9,10]. Oral health may also impact other domains of daily living including oral health-related quality of life (OHRQoL). OHRQoL has been established as an important outcome to measure in the oral health literature [11]. A previous systematic review summarized oral health research among individuals with hearing impairments and visual impairments [12]. Since then, the body of evidence on oral health in individuals with sensory impairments has continued to expand. New studies have investigated oral health outcomes beyond traditional clinical outcomes to include variables such as treatment needs, OHRQoL, traumatic dental injuries, salivary, and microbiological characteristics [13,14,15,16,17]. This systematic review aimed to evaluate the evidence on oral health status, dental treatment needs, oral health-related quality of life, and other clinically relevant oral health outcomes in individuals with hearing and visual impairments.

## 2. Materials and Methods

This systematic review was reported in accordance with the Preferred Reporting Items for Systematic Reviews and Meta-Analyses (PRISMA 2020) guidelines [18]. The completed PRISMA 2020 Checklist is provided as Supplementary File S1. The review question was formulated according to the Population, Intervention, Comparator, Outcome (PICO) framework. As only observational studies were included, the intervention component was interpreted as the exposure (hearing impairment and/or visual impairment). The PICO framework used to formulate the review question is summarized in Table 1.

### 2.1. Search Strategy

A systematic literature search was conducted in the bibliographic databases Ovid MEDLINE, Ovid Embase, and Web of Science from database inception to 8 January 2026, in collaboration with a medical information specialist.

The search strategy combined controlled vocabulary terms, such as Medical Subject Headings (MeSH) and Emtree, and free-text terms related to oral health and sensory impairments, including hearing impairment, deafness, hard of hearing, visual impairment, blindness, and low vision. The full search strategies for all databases are provided as Supplementary File S2.

The reference lists of the included studies and relevant review articles were screened to identify additional eligible publications. No language restrictions were applied to the search.

Duplicate records were removed using EndNote X21 (Clarivate, Philadelphia, PA, USA), following the Amsterdam Efficient Deduplication (AED) [19] and Bramer [20] methods.

### 2.2. Selection Process

Two reviewers (T.K. and L.N.F.) independently screened all potentially relevant titles and abstracts for eligibility using the Rayyan web application (Rayyan Systems Inc., Cambridge, MA, USA; web version) [21]. If necessary, full-text articles were retrieved and assessed for eligibility. Differences in judgement were resolved through discussion and consensus.

The predefined inclusion and exclusion criteria are summarized in Table 2.

When studies included additional comparison groups, only data from participants with hearing impairment (HI) and/or visual impairment (VI) were extracted and analyzed. When studies reported both HI and VI participants, subgroup-specific data were extracted separately whenever available. Studies reporting combined HI/VI populations without separate subgroup analyses were retained and synthesized separately from the HI and VI subgroups.

### 2.3. Data Extraction

Data extraction was performed by one reviewer (T.K.) using a standardized data extraction form. The extracted data were subsequently checked for accuracy and completeness by a second reviewer (L.N.F.). Any discrepancies were resolved through discussion.

Data were extracted from each included study using a structured data extraction table. The following information was collected: author and year of publication, country, study design, type of sensory impairment, sample size, participant age characteristics, oral health assessment methods, and reported oral health outcomes.

The primary oral health outcomes included oral hygiene measures, periodontal measures, dental caries outcomes, and treatment needs.

Additional outcomes included oral health-related quality of life, traumatic dental injuries, oral health behaviours, dental attendance patterns, access-to-care indicators, salivary findings, microbiological findings, functional oral outcomes, and other clinically relevant oral health findings.

Age ranges and mean ages were extracted as reported by the original authors. When age was presented in categories, the overall age range was derived from the reported minimum and maximum category limits.

Data were extracted as reported in the original articles and subsequently standardized for presentation to improve comparability across studies, without altering the original meaning or numerical values.

### 2.4. Risk of Bias Assessment

The methodological quality of the included studies was assessed using the appropriate Joanna Briggs Institute (JBI) Critical Appraisal Checklists according to study design [22,23].

The assessment considered study design, sample characteristics, sampling strategy, clarity of the sensory impairment group, validity and reliability of oral health assessment methods, use of standardized clinical indices, presence or absence of control groups, adjustment for potential confounding factors, and appropriateness of statistical analysis.

No formal numerical quality score was assigned. Instead, methodological quality was appraised narratively to identify strengths and limitations of the included studies and to support interpretation of the findings during data extraction and synthesis.

Risk of bias assessment was performed independently by two reviewers (T.K. and L.N.F.), with disagreements resolved through discussion.

### 2.5. Certainty of Evidence Assessment

The certainty of the evidence for the primary oral health outcomes was assessed using the Grading of Recommendations Assessment, Development and Evaluation (GRADE) approach [24]. The certainty of evidence was evaluated across the domains of risk of bias, inconsistency, indirectness, and imprecision.

Because the evidence was derived primarily from observational studies, the certainty of evidence started at a low level and was downgraded where appropriate according to the GRADE methodology.

Separate GRADE evidence profiles were prepared for hearing impairment and visual impairment.

### 2.6. Data Synthesis

Due to substantial clinical and methodological heterogeneity across the included studies with respect to study design, participant characteristics, outcome definitions, and assessment methods, a quantitative synthesis (meta-analysis) was not considered appropriate.

Results were organized according to type of sensory impairment and oral health outcomes. Studies involving HI and VI were described separately where possible. Combined HI/VI studies were synthesized as a separate category.

As no meta-analysis or statistical re-analysis of the primary data was performed, no independent threshold for statistical significance was applied. Instead, statistical significance was interpreted according to the original studies. Where available, reported *p* values and other measures of statistical significance were incorporated into the narrative synthesis.

## 3. Results

### 3.1. Study Selection

**Figure 1:**
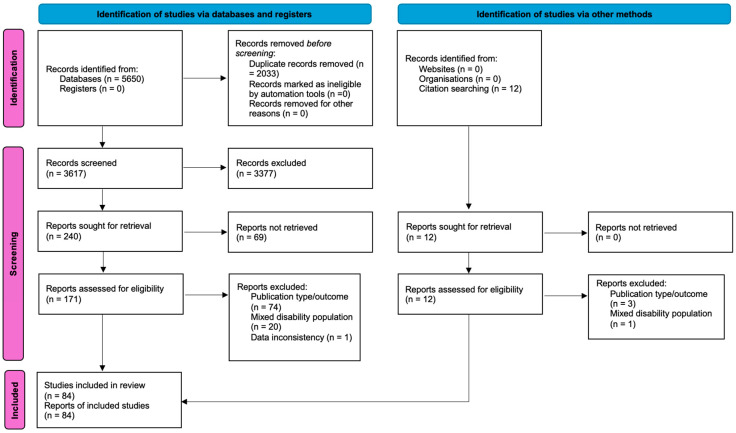
Preferred Reporting Items for Systematic Reviews and Meta-Analyses (PRISMA) 2020 flow diagram of the study selection process.

The literature search identified 5650 records. After duplicate removal, 3617 records remained for screening, of which 3377 were excluded. A total of 240 reports were sought for full-text retrieval. Of these, 69 could not be retrieved, leaving 171 reports assessed for eligibility. Following full-text assessment, 95 reports were excluded because they did not meet the predefined eligibility criteria, including intervention studies [25,26,27,28], studies including participants with disabilities without separately reporting data for hearing or visual impairment [29,30,31,32], studies reporting only knowledge, awareness, attitudes, or oral hygiene practices without objective clinical oral health outcomes [33,34,35], or due to inconsistencies in the reported data [36]. In addition, reference screening identified eight further eligible studies, resulting in a total of 84 studies included in the qualitative synthesis. The study selection process is presented in Figure 1.

### 3.2. Study Characteristics

The included studies involved individuals with HI, VI, or both. The studies were conducted across multiple countries worldwide. Most studies focused on children and adolescents. Sample sizes varied considerably across studies. Detailed characteristics of the included studies are presented in Table 3.

### 3.3. Risk of Bias

The methodological quality of the included studies was assessed using the appropriate Joanna Briggs Institute (JBI) Critical Appraisal Checklists according to study design. Overall, 18 of the 84 included studies (21.4%) were classified as having a low risk of bias, 45 (53.6%) as having a moderate risk of bias, and 21 (25.0%) as having a high risk of bias. A summary of the risk of bias assessment is presented in Table 4.

### 3.4. Oral Health Outcomes in Individuals with HI

Reported outcomes in HI individuals included oral hygiene measures, periodontal status, dental caries outcomes, dental needs, and other oral health-related findings. Detailed findings from the included studies are presented in Table 5.

#### 3.4.1. Oral Hygiene and Periodontal Status

Oral hygiene findings among individuals with HI were variable; however, poor oral hygiene was commonly reported across studies. In studies that classified oral hygiene status categorically, the prevalence of poor oral hygiene ranged from 31.5% to 58.0% [13,75,77,105,107]. Elevated Oral Hygiene Index—Simplified (OHI-S), plaque index (PI), and visible plaque index (VPI) values further indicated suboptimal oral hygiene in several populations [10,42,61,88,97].

Several studies identified inadequate oral hygiene practices, including low toothbrushing frequency, limited use of dental floss, irregular dental attendance, and poor oral health awareness [57,77,80,103]. Poorer oral hygiene practices and higher caries experience were reported in several studies [48,72,77]. In addition, Azfar et al. reported that 59.4% of participants brushed their teeth only once daily, 57.5% had never visited a dentist, and frequent consumption of sugary foods and beverages was common [47]. Ghannam et al. [14] reported significant positive correlations between hearing loss severity and both plaque index (PI; *p* < 0.001) and gingival index (GI; *p* < 0.001). Tugeman et al. [110] additionally reported mature plaque in 50.8% of participants and acid-producing plaque in 14.8% of participants. They also reported higher dental plaque maturity scores among children with hearing impairment than among controls (*p* < 0.001).

Periodontal problems were frequently reported among HI individuals. Gingivitis was among the most common findings, with prevalence ranging from 15.8% to 80.5% [44,80,91,103,105]. Gingival bleeding was also commonly reported [10,13,72,81,112], reaching levels of up to 82.4% [89]. Similarly, bleeding on probing (BOP) was reported in 18.3–43.6% of participants [49,65,107].

Calculus accumulation was frequently observed, affecting between 11.1% and 78.5% of participants [10,63,65,72]. Periodontal pockets and periodontal disease were additionally reported in several studies [10,13,63,81,107], while only 16.9% of participants were classified as periodontally healthy in the study by Goud et al. [10].

These findings indicate poor oral hygiene and periodontal problems among individuals with HI.

#### 3.4.2. Caries Experience

Caries experience was the most frequently reported oral health outcome and was assessed in almost all included studies involving HI individuals. Most studies reported high caries burden, although prevalence varied considerably between populations and age groups. High caries prevalence was observed across both paediatric and adult HI populations. Caries prevalence ranged from 19.3% to 95.7%, with most studies reporting prevalence above 50% [72,84,89] and several reporting values exceeding 80% [14,41,50,61,113].

Several comparative studies demonstrated poorer caries outcomes among HI participants than among controls. Higher caries prevalence, lower proportions of caries-free individuals, and higher caries experience were consistently reported in HI individuals than in control groups [48,68,113]. For example, Kar et al. [68] reported higher caries prevalence among children with HI than healthy controls (*p* < 0.05), while Vyavahare et al. [113] found higher DMFT values among children with HI than controls (6.80 ± 6.00 vs. 1.26 ± 1.72, *p* < 0.001). Bakardjiev [48] additionally reported higher DMF scores among children with severe hearing impairment than among those with mild-to-moderate hearing impairment or healthy controls (*p* < 0.05). Similarly, Samal et al. [89] reported a significant association between hearing loss severity and the presence of dental caries (*p* = 0.027).

In several studies, decayed teeth constituted the largest component of the DMFT/dmft index, whereas the proportion of filled teeth was generally low [62,97]. Mean DMFT and dmft values varied considerably across studies.

These findings indicate a high burden of dental caries among individuals with HI.

#### 3.4.3. Other Oral Findings

Additional oral findings among HI individuals included orthodontic anomalies, traumatic dental injuries (TDI), salivary findings, reduced oral health-related quality of life (OHRQoL), and functional oral limitations.

Orthodontic findings included Class II and Class III malocclusions, spacing, tooth rotation, crowding, impacted teeth, and other occlusal anomalies [13,44,50,105]. Severe malocclusion was reported in 13.4% of participants by Buela et al. [13]. Brozd-Topolko et al. [50] reported that orthodontic anomalies affected 57.8% of HI children and were more prevalent than in healthy controls (*p* < 0.05).

Traumatic dental injuries (TDI) were reported by Singh et al. [100], with a prevalence of 40.8%.

Salivary findings were described in several studies as reduced salivary flow rate, lower salivary pH, and reduced salivary buffering capacity [37,113].

Reduced OHRQoL was reported in both studies evaluating quality-of-life outcomes. Ghannam et al. [14] reported significant positive correlations between paediatric OHRQoL and DMFT (*p* < 0.001), dmft (*p* < 0.001), plaque index (PI; *p* < 0.001), and gingival index (GI; *p* = 0.038), while Singh et al. [100] reported less favourable OHRQoL among HI children. Functional oral problems were reported in several studies and were described in both HI children and HI adults. Pradhan et al. [80] found chewing difficulties in 22.1% of HI children, whereas Kim et al. [70] reported fewer remaining natural teeth (*p* < 0.001), poorer maxillary and mandibular prosthesis status (both *p* < 0.001), poorer chewing function (*p* < 0.001) and poorer speaking function (*p* < 0.001) among adults with hearing loss than among controls. These findings illustrate a broad spectrum of additional oral health problems among individuals with HI.

#### 3.4.4. Dental Needs

A wide range of dental needs was identified among HI individuals. Restorative treatment was the most frequently reported need, particularly one- and two-surface restorations [10,37,62,103]. Additional needs included extractions, pulp therapy, fissure sealants, topical fluoride applications, and oral health education [10,37,62].

In studies reporting overall treatment requirements, 85.6–91.7% of participants required some form of dental treatment [42,91,103].

Unmet dental needs were also evident. Djeri et al. [57] reported that 25.8% of participants in Bosnia and Herzegovina had never visited a dentist. Substantially higher levels of unmet dental care were reported in studies from India, where 80.3% of participants had never received dental care [103], none of the children had ever visited a dentist [87], and 90.1% had never received dental care or visited a dentist [89].

Across studies, recommendations were generally consistent. Frequently recommended measures included preventive oral health programmes, oral health education, caregiver involvement in oral hygiene practices, improved access to dental services, and reduction in communication barriers between dental professionals and HI individuals [14,65,110]. Most authors emphasized preventive approaches and improved access to oral healthcare as key priorities for this population.

Taken together, these findings indicate high treatment needs and considerable unmet dental care among individuals with HI.

### 3.5. Oral Health Outcomes in Individuals with VI

Reported outcomes in VI individuals included oral hygiene measures, periodontal status, dental caries outcomes, dental needs, and other oral health-related findings. Detailed findings from the included studies are presented in Table 6.

#### 3.5.1. Oral Hygiene and Periodontal Status

Oral hygiene findings among VI individuals were variable; however, fair-to-poor oral hygiene was commonly reported across studies. In studies that classified oral hygiene status categorically, the prevalence of poor oral hygiene ranged from 0.4% to more than 70% [13,55,69,96,112]. OHI-S, plaque index, and visible plaque index values further indicated suboptimal oral hygiene in several populations [10,43,69,78,106].

Comparative studies generally indicated poorer oral hygiene and periodontal health among VI individuals than among sighted controls [15,43,54]. AlSadhan et al. [43] reported higher oral hygiene index (OHI) scores (*p* = 0.001), plaque index (PI) scores (*p* < 0.0001), and gingival index (GI) scores (*p* = 0.001) among children with VI than among sighted controls. Hazra et al. [15] similarly reported poorer oral hygiene (*p* < 0.001) and gingival health (*p* < 0.001) among children with VI than among controls.

Periodontal problems were commonly reported among VI individuals. Gingivitis prevalence ranged from 34.4% to 88.1% [74,96], while several studies additionally reported gingival inflammation or moderate-to-severe gingivitis [15,49]. Gingival bleeding ranged from 8.8% to 94.7% [10,62,81,112]. Similarly, bleeding on probing (BOP) ranged from 42.9% to 79.2% [49,95,108].

Dental calculus accumulation was also frequently observed, with prevalence ranging from 13.4% to 86.9% [10,49,62,73]. Periodontal pockets, attachment loss, and periodontal disease were additionally reported across several studies, with periodontal pockets affecting up to 81.2% of participants [62,81,108].

These findings indicate compromised oral hygiene and periodontal health among individuals with VI.

#### 3.5.2. Caries Experience

Caries experience was one of the most frequently reported oral health outcomes among VI individuals and was assessed in nearly all included studies. Caries prevalence varied considerably across populations and age groups, ranging from 15.0% to 98.5%. Prevalence values above 80% were reported in many studies [16,50,66,85,96]. The highest prevalence was reported by Shetty et al. [96], in which 98.5% of participants were affected by caries, followed by Brozd-Topolko et al. [50] and Feng et al. [16], both reporting prevalence exceeding 90%.

Several comparative studies demonstrated poorer caries outcomes among VI individuals than among sighted controls. Higher caries experience was reported in VI participants in studies comparing DMFT, DMFS, or caries prevalence between groups [15,43,51]. For example, AlSadhan et al. [43] reported higher DMFS scores among children with VI than among sighted controls (*p* = 0.03), while Hazra et al. [15] reported higher caries experience among children with VI than among controls (*p* < 0.001).

Using a Cariogram-based assessment, Daryani et al. [54] reported a lower chance of avoiding future caries among VI children (41%) than among controls (74%), indicating a higher estimated caries risk in the VI group.

Several studies highlighted a high proportion of untreated decay. Rajab and Mismar [85] reported that untreated caries represented the predominant component of overall caries experience, while Lee et al. [71] found that untreated decay accounted for approximately 80% of the DMFT index. These findings illustrate a high prevalence of dental caries and a considerable proportion of untreated decay among individuals with VI.

These findings indicate a high burden of dental caries among individuals with VI.

#### 3.5.3. Other Oral Findings

Additional oral findings among VI individuals included orthodontic anomalies, traumatic dental injuries (TDI), altered salivary and microbiological findings, OHRQoL measures, and functional oral limitations.

Orthodontic anomalies were frequently reported among VI individuals. Brozd-Topolko et al. [50] reported orthodontic anomalies in 69.9% of VI children, more often than in healthy controls (*p* < 0.05). Other reported findings included malocclusion (49.5%) reported by Liu et al. [73], maxillary crowding (40%) and mandibular crowding (48%), reported by Darwita et al. [53]. Severe malocclusion was additionally reported in 37.2% of participants by Buela et al. [13].

TDI were frequently reported among VI individuals, with prevalence ranging from 4.6% to 50.6% [67,85,99,102]. Comparative findings also suggested a higher prevalence of TDI among VI individuals than among sighted controls [76]. Srivastava et al. [102] additionally found a higher prevalence of TDI among participants with acquired VI than among those with congenital VI (*p* = 0.04).

Salivary and microbiological findings were described in several studies. Reduced salivary flow rate, lower salivary buffering capacity, lower total antioxidant capacity, altered salivary biomarkers, and increased salivary streptococcal counts were reported among VI participants [15,39,46,54]. Hazra et al. [15] additionally reported lower salivary total antioxidant capacity (TAC) among children with VI than among controls (*p* < 0.001). In addition, Feng et al. [16] described alterations in the salivary microbiota associated with gingivitis and caries.

Reduced OHRQoL was reported in studies evaluating quality-of-life outcomes. Singh et al. [99] reported unfavourable OHRQoL among VI children, particularly among those with dentofacial anomalies, dental caries, and TDI, while Tagelsir et al. [106] reported higher Child Oral Impacts on Daily Performance (Child-OIDP) scores among children with complete VI than among those with partial VI.

Functional oral limitations were reported among VI adults. Samnieng et al. [90] observed difficulties with chewing, swallowing, speaking, and tasting. Additional oral findings included tongue thrusting and mouth breathing, as reported by Goud et al. [10].

These findings illustrate a broad spectrum of additional oral health outcomes among individuals with VI.

#### 3.5.4. Dental Needs

Dental needs among VI individuals included restorative treatment, extractions, pulp therapy, prosthetic treatment, and preventive care.

One-surface restorations were among the most frequently reported treatment needs, affecting 35.9–95.0% of participants in studies conducted in India [10,13,79,104]. Additional treatment needs included pulp therapy (13–19.4%) and extractions (16–22.3%) [63,79,104,112].

In studies reporting overall treatment requirements, 40.0–71.0% of participants required dental treatment, while 20.0–28.0% required immediate treatment [53,64]. Tagelsir et al. [106] reported urgent treatment needs in 25.3% of participants, with more than 90% of these needs related to deep caries or pulp involvement.

Unmet treatment needs were also described in several populations. Rezaei et al. [88] reported a high unmet treatment need index (UTN 0.76 ± 0.34) in Iran. Sharififard et al. [94] reported that 62.3% of adolescents with VI had at least one untreated decayed tooth, while Sharififard et al. [95] reported unrestored caries in 57.4% of primary teeth and 34.0% of permanent teeth among children with VI. In India, Shetty et al. [96] reported a high proportion of untreated decay, while Suresan et al. [104] reported high unmet dental treatment needs. Samnieng et al. [90] additionally reported that 38.2% of participants required upper and lower prostheses.

Across studies, recommendations were generally consistent. Most authors emphasized oral health education, preventive programmes, regular dental attendance, caregiver support, and improved accessibility of dental services for individuals with VI [6,9,43,81].

Taken together, these findings indicate high treatment needs and considerable unmet dental care among individuals with VI.

### 3.6. Oral Health Outcomes in Studies Reporting Hearing and Visual Impairments Without Separate Subgroup Analyses

One study reported oral health outcomes for participants with hearing and visual impairments without separate subgroup analyses [38]. The study reported poor oral hygiene (56%), high caries prevalence (77.3%), multiple dental treatment needs, and limited utilization of dental services, with 60% of participants never having visited a dentist. Oral health problems additionally affected eating, sleeping, and studying in some participants. Due to the absence of separate subgroup analyses, these findings could not be attributed specifically to either HI or VI. Detailed findings are presented in Table 7.

### 3.7. Certainty of Evidence (GRADE)

The certainty of evidence was assessed using the GRADE approach. Across both hearing and visual impairment, the certainty of evidence was rated as very low for all assessed outcomes, including dental caries, oral hygiene, periodontal status, and treatment needs. Downgrading of the certainty of evidence was primarily driven by concerns regarding the risk of bias and inconsistency across the included studies, with additional downgrading for imprecision where applicable. The detailed GRADE evidence profiles are presented in Table 8a,b.

## 4. Discussion

The findings of this review highlight the multifactorial nature of oral health outcomes among individuals with sensory impairments. Oral health problems were prevalent in both populations included in the review; however, there was substantial heterogeneity across study populations, settings, and outcome measures. Some of this heterogeneity may be explained by genuine differences in determinants of oral health across populations, including differences in access to healthcare services, caregiver support, oral health literacy, and severity of sensory impairment.

The periodontal findings observed across the included studies suggest that oral hygiene behaviours, oral health knowledge, and caregiver support may play an important role in maintaining periodontal health among individuals with sensory impairments. Several studies identified associations between oral health outcomes and factors such as toothbrushing frequency, parental education, oral health awareness, and caregiver involvement [52,72,74,84]. These observations highlight the potential importance of educational and caregiver-related factors in oral health among individuals with sensory impairments.

Direct comparisons between populations with hearing and visual impairments were limited, and the available evidence was not entirely consistent. Goud et al. [10] reported a higher prevalence of gingival bleeding and a lower proportion of periodontally healthy individuals among participants with VI than among participants with HI. Similar observations were reported by Tefera et al. [108] and Reddy and Sharma [86], who found poorer oral hygiene and periodontal outcomes among individuals with VI. In contrast, Jain et al. [63] reported a higher proportion of periodontally healthy individuals with VI than those with HI.

This limited and conflicting comparative evidence suggests that differences in oral health outcomes may be influenced by multiple factors and cannot be attributed to the type of sensory impairment alone. However, the available evidence suggests that the underlying determinants of oral health problems may differ between impairment groups. Communication difficulties were commonly identified in studies involving individuals with HI, whereas dependence on others for daily activities and access to adapted oral health information appeared to be more relevant among individuals with VI.

Dental caries remains one of the most important oral health concerns among individuals with sensory impairments. Similar to periodontal outcomes, caries experience appears to be influenced by a range of behavioural and environmental factors. Several studies reported associations between higher caries experience and lower toothbrushing frequency, lower parental education, limited oral health awareness, dietary habits, caregiver involvement, and reduced access to preventive and restorative dental services [9,45,52,72,82].

Daryani et al. [54] further reported a significantly lower predicted chance of avoiding future caries among children with VI than among controls (41% vs. 74%), indicating a higher estimated caries risk in this population.

In addition, Puteri et al. [83] reported an association between oral health knowledge and caries experience, highlighting the potential importance of oral health literacy in caries prevention.

Comparative evidence regarding caries outcomes was also limited. Sanjay et al. [92] reported higher DMFT values among blind children than among children with HI, whereas Jain et al. [63] reported higher DMFT/dft values among participants with HI. Although these findings provide limited insight into potential differences in caries experience between sensory impairment groups, they indicate that dental caries represents a substantial oral health burden in both populations.

Another important finding was the apparent relationship between the severity of hearing impairment and oral health outcomes. Greater severity of hearing loss was associated with poorer oral health outcomes, including higher caries experience, increased plaque accumulation, and greater gingival inflammation across several studies [48,61,89]. This association suggests that impairment severity itself may contribute to some of the differences observed in oral health outcomes, in addition to behavioural and environmental factors. Severity of hearing loss may also partly explain the substantial heterogeneity observed across HI populations, as most studies did not report hearing loss severity or incorporate it into the interpretation of their findings.

Another important finding was the consistently high level of untreated decay. In several studies, decayed teeth accounted for a much larger proportion of the DMFT/dmft index than filled teeth. Jain et al. [63] reported that 83.9% of participants had decayed teeth, whereas only 7.1% had filled teeth. Similarly, Lee et al. [71] found that untreated decay constituted approximately 80% of the overall DMFT index among children with VI. High levels of untreated caries were also reported in Iran [88,94,95], Jordan [85], and India [96,104]. In contrast, Dumančić et al. [9] reported a median DMFT of 17.0 and a median D component of only 1.0 among adults with VI in Croatia, suggesting that most caries experience reflected previously restored or extracted teeth rather than untreated decay. These discrepant findings indicate that the oral disease burden experienced by individuals with sensory impairments may be influenced not only by impairment-related factors but also by healthcare system characteristics and access to dental care.

Restorative treatment was the most frequently reported dental need, although extractions, pulp therapy, preventive interventions, and, in some populations, prosthetic treatment were also required [90,94,95,104,106]. Particularly noteworthy was the high unmet treatment need index reported by Rezaei et al. [88], suggesting that a large proportion of treatment needs remained unresolved despite the considerable burden of oral disease. Together, these findings indicate that barriers to accessing dental care remain common among individuals with sensory impairments.

Several studies reported oral health outcomes beyond oral hygiene, periodontal status, and dental caries. TDI were reported predominantly among individuals with VI [67,85,99,102,106]. Reduced visual perception may increase the likelihood of falls, collisions, and other environmental accidents, thereby contributing to a greater risk of dental trauma. This interpretation is supported by the systematic review and meta-analysis by Devi et al. [114], which identified VI as an important risk factor for TDI among individuals with special healthcare needs. More recently, Reddy et al. [17] demonstrated that children with VI had approximately five-fold higher odds of experiencing TDI than children with hearing and speech impairments. Furthermore, Srivastava et al. [102] reported a higher prevalence of TDI among individuals with acquired VI than among those with congenital VI, suggesting that adaptation to visual loss may also influence trauma risk.

Altered salivary characteristics were reported in several studies involving individuals with VI, including reduced salivary flow rate, lower buffering capacity, reduced total antioxidant capacity, altered salivary biomarkers, and changes in growth factor expression [15,39,54]. These findings may be clinically relevant because saliva plays a central role in maintaining oral homeostasis and protecting against both dental caries and periodontal disease through its buffering, antimicrobial, antioxidant, and remineralizing properties [115,116,117].

Similarly, microbiological studies suggested differences in the oral microbial environment of individuals with sensory impairments. Daryani et al. [54] identified salivary and microbial factors as predictors of caries risk, while Feng et al. [16] described alterations in the salivary microbiota associated with gingivitis and caries in VI individuals.

Recent microbiome research has demonstrated alterations in the salivary microbial community of children with HI, further supporting the hypothesis that biological factors may contribute to oral health disparities in this population [118]. Previous studies have demonstrated that salivary biomarkers may reflect oral inflammatory processes, including gingival inflammation [119,120]. These findings indicate that biological mechanisms may represent an additional component of oral health in individuals with sensory impairments and warrant further investigation.

Several studies additionally reported reduced OHRQoL, while others described functional oral limitations, including difficulties with chewing, tasting, swallowing, and speaking [14,90,99,100]. These findings suggest that the impact of oral health problems extends beyond clinical disease and may affect the daily functioning and overall well-being of individuals with hearing and visual impairments.

### 4.1. Strengths and Limitations

This review expands current knowledge on oral health in individuals with sensory impairments by synthesizing evidence across a broad range of oral health outcomes in both hearing and visual impairment populations. Including both sensory impairment groups enabled comparison of oral health outcomes and provided insight into both shared and impairment-specific oral health challenges and treatment needs. Furthermore, a comprehensive search strategy was developed in collaboration with a medical information specialist and captured a broad spectrum of oral health outcomes. In addition to oral hygiene, periodontal health, and dental caries, this review also evaluated treatment needs, TDI, salivary and microbiological findings, functional oral limitations, and OHRQoL, providing a comprehensive overview of oral health in individuals with sensory impairments.

Several limitations should be considered when interpreting the findings of this review. First, substantial heterogeneity was observed across studies with regard to participant characteristics, age groups, outcome definitions, and assessment methods, which limited direct comparisons between studies. Given these methodological and clinical differences, the results were synthesized narratively rather than quantitatively. The observed heterogeneity also suggests that oral health outcomes may be influenced not only by sensory impairment itself, but also by differences in study populations, healthcare settings, and assessment methods. Therefore, caution is warranted when generalizing these findings to all individuals with sensory impairments. In addition, many studies were based on school- or institution-based samples and may therefore not fully represent the broader population of individuals with sensory impairments. Furthermore, most included studies were cross-sectional, limiting conclusions regarding potential causal relationships between oral health outcomes and associated factors. The severity of sensory impairment was also inconsistently reported and was rarely considered in the analyses, making it difficult to assess its potential influence on oral health outcomes. Finally, most studies were conducted outside Europe, resulting in limited representation of European populations and potentially restricting the generalizability of the findings to European healthcare settings.

### 4.2. Future Research

Future research should prioritize longitudinal and population-based studies and improve comparability through more standardized assessment and reporting of oral health outcomes. Further comparative studies involving individuals with hearing and visual impairments are needed to improve understanding of potential differences in oral health outcomes between sensory impairment groups. Additional research on salivary, microbiological, and other biological factors may also contribute to a better understanding of oral health in these populations. Participant characteristics, healthcare accessibility, caregiver support, and severity of sensory impairment were rarely examined in the included studies. Greater consideration of these factors in future research may provide a more comprehensive understanding of oral health in individuals with sensory impairments. In particular, severity of sensory impairment should be reported and evaluated more consistently, as available evidence suggests that it may be an important predictor of oral health outcomes.

## 5. Conclusions

Poor oral hygiene, periodontal problems, dental caries, and unmet dental treatment needs were among the most frequently reported findings in individuals with hearing and visual impairments. In addition to sensory impairment, behavioural and environmental factors, including oral hygiene practices, caregiver involvement, parental education, and access to dental services, may also influence oral health outcomes in this population. Future well-designed studies using standardized outcome measures are needed to strengthen the evidence base and support the development of targeted oral healthcare strategies. These findings highlight the need for preventive strategies, accessible oral health education, and improved access to dental care for individuals with hearing and visual impairments.

## Figures and Tables

**Table 1:** PICO framework used to formulate the review question.

PICO Component	Description
Population (P)	Individuals of any age with hearing impairment and/or visual impairment
Intervention/Exposure (I/E)	Hearing impairment and/or visual impairment (observational studies; no intervention was applicable)
Comparator (C)	Individuals without sensory impairments, where available
Outcome (O)	Oral health status, dental treatment needs, oral health-related quality of life, and other clinically relevant oral health outcomes

**Table 2:** Eligibility criteria for study selection.

Inclusion Criteria	Exclusion Criteria
Observational studies (cross-sectional, cohort, or case–control)	Reviews, systematic reviews, meta-analyses, editorials, opinion papers
Human participants of any age with hearing impairment (HI), visual impairment (VI), or both	Studies focusing only on knowledge, attitudes, access to care, or educational interventions without reporting oral health outcomes
Original research reporting primary data	Animal studies
Reported oral health outcomes, treatment needs, oral health-related quality of life, or other clinically relevant oral health findings	Case reports or case series with <20 participants
Clearly described oral health assessment methods (e.g., clinical examinations, standardized indices, validated questionnaires)	Mixed disability populations in which HI/VI data could not be extracted separately
Full-text available	Unclear or insufficient description of the sensory impairment group
Published in English, Dutch, or Croatian	Duplicate datasets
	Full text unavailable at the time of assessment
	Published in languages other than English, Dutch, or Croatian

**Table 3:** Characteristics of included studies.

Study (Country)	Design	Sensory Type	NpI [*n*]	Nc [*n*]	Age M ± SD (Range)	Male Gender [*n* (%)]
Abd. Rahman et al., 2015 (Malaysia) [37]	CS	HI	63	NR	11.5 ± 2.4 (7–14)	29 (46.2%)
Agbor et al., 2020 (Cameroon) [38]	CS	HI + VI *	180	NR	(5–35)	77 (43.0%)
Ajeel & Diab, 2024 (Iraq) [39]	CS	VI	70	110	(8–10)	NR
Al-Maweri et al., 2015 (Yemen) [40]	CS	HI	92	NR	10.18 ± 2.41 (6–14)	62 (67.4%)
		VI	50		9.84 ± 2.26 (6–14)	50 (100.0%)
Al-Qahtani & Wyne, 2004 (Saudi Arabia) [41]	CS	HI	80	NR	(6–12)	0 (0%) †
		VI	29	NR	(6–12)	0 (0%) †
Alkahtani et al., 2019 (Saudi Arabia) [42]	CS	HI	146	NR	≥18	41 (28.1%)
AlSadhan et al., 2017 (Saudi Arabia) [43]	CS	VI	79	83	9.8 ± 2.0 (6–12)	0 (0%) †
Alyami et al., 2022 (Saudi Arabia) [44]	CS	HI	116	NR	(5–16)	60 (51.7%)
Amrollahi et al., 2020 (Iran) [45]	CS	VI	50	NR	10.3 ± 1.7 (8–14)	27 (54%)
Anand et al., 2021 (India) [46]	CS	VI	180	NR	12.0 ± 2.6 (7–20)	NR
Azfar et al., 2018 (Pakistan) [47]	CS	HI	106	NR	12.88 ± 2.59 (5–15)	64 (60.4%)
Bakardjiev, 2025 (Bulgaria) [48]	CS	HI	37	21	(7–10)	NR
Bimstein et al., 2014 (USA) [49]	CC	HI	85	119	NR ‡	NR ‡
		VI	35	119	NR ‡	NR ‡
Brozd-Topolko et al., 1988 (Croatia) [50]	CS	HI	83	166	(6–17)	46 (55.4%)
		VI	83	166	(6–17)	49 (59.0%)
Buela et al., 2024 (India) [13]	CS	HI	112	NR	(5–34)	40 (35.7%)
		VI	78	NR	(5–34)	35 (44.9%)
Cunha & Bizarra, 2023 (Portugal) [51]	CS	VI	34	34	11.1 ± 3.4 (6–18)	NR
da Cunha et al., 2015 (Brazil) [52]	CS	VI	52	NR	25.6 ± 7.9	NR
Darwita et al., 2024 (Indonesia) [53]	CS	VI	25	NR	(6–12)	11 (44%)
Daryani et al., 2014 (India) [54]	CS	VI	60	60	(7–36)	NR
Debnath et al., 2025 (India) [55]	CS	VI	260	500	(7–15)	155 (59.6%)
Dhanvanth et al., 2020 (India) [56]	CS	VI	20	NR	NR	12 (60.0%)
Djeri et al., 2013 (Bosnia & Herzegovina) [57]	CS	HI	66	NR	(3–18)	36 (54.5%)
Dogra et al., 2019 (India) [58]	CS	VI	1000	NR	12	NR
Dumančić et al., 2025 (Croatia) [9]	CS	VI	85	85	(18–98)	43 (50.6%)
Feng et al., 2025 (China) [16]	CS	VI	101	NR	(6–16)	NR
Gaçe et al., 2014 (Albania) [59]	CS	HI	147	NR	NR #	NR
		VI	91	NR	NR #	NR
Gaikwad et al., 2022 (India) [60]	CS	VI	400	NR	21.2 (15–30)	NR
Ghannam et al., 2024a (Syria) [14]	CS	HI	90	NR	(6–12)	NR
Ghannam et al., 2024b (Syria) [61]	CS	HI	90	NR	(6–12)	NR
Goud et al., 2021 (India) [10]	CS	HI	207	NR	(6–24)	121 (58.4%)
		VI	77	NR	(6–24)	37 (48.0%)
Hazra et al., 2022 (India) [15]	CS	VI	50	50	9.7 ± 2.0 (6–11)	26 (52%)
Jain et al., 2008 (India) [62]	CS	HI	127	NR	(5–22)	NR
Jain et al., 2013 (India) [63]	CS	HI	297	NR	(4–23)	NR
		VI	201	NR	(4–23)	NR
Jawahar & Maragathavalli, 2022 (India) [64]	CS	VI	207	NR	(21–80)	133 (64.3%)
Jnaneswar et al., 2017 (India) [65]	CS	HI	540	NR	(5–15)	262 (48.5%)
John et al., 2017 (India) [66]	CS	VI	404	NR	22.6 (15–30)	207 (51.2%)
Kannappan et al., 2023 (India) [67]	CS	VI	130	NR	(6–12)	73 (56.2%)
Kar et al., 2016 (India) [68]	CC	HI	236	272	(6–12)	138 (58.5%)
Khan et al., 2020 (Malaysia) [69]	CS	VI	91	NR	15.6 ± 1.7 (13–17)	56 (61.5%)
Kim et al., 2024 (South Korea) [70]	CS	HI	1384	1738	≥40	688 (55.5%)
Lee et al., 2024 (Hong Kong) [71]	CS	VI	73	NR	12.9 ± 4.7 (6–21)	42 (57.5%)
Li et al., 2023 (China) [72]	CS	HI	56	NR	(6–31)	29 (51.8%)
		VI	118	NR	(6–31)	71 (60.2%)
Liu et al., 2019 (China) [73]	CS	VI	103	NR	15.9 ± 4.3 (6–20)	70 (68.0%)
Macharia et al., 2023 (Kenya) [74]	CS	VI	159	NR	13.9 ± 2.3 (10–19)	85 (53.5%)
Mishra et al., 2021 (India) [75]	CS	VI	277	NR	(6–15)	NR ‡
			97	NR	(6–15)	NR ‡
Mohd-Dom et al., 2010 (Malaysia) [76]	CS	VI	39	NR	NR	17 (43.6%)
Mungan Durankaya et al., 2025 (Turkey) [77]	CS	HI	40	44	(6–13)	15 (37.5%)
Ozdemir-Ozenen et al., 2012 Turkey) [78]	CS	VI	50	50	7.5 ± 0.5 (6–10)	24 (48%) §
Parkar et al., 2014 (India) [79]	CS	VI	160	NR	14.66 ± 3.27 (8–22)	100 (62.5%)
Pradhan et al., 2015 (Nepal) [80]	CS	HI	81	NR	≥18	NR
Prasad et al., 2018 (India) [81]	CS	HI	460	NR	NR ‡	NR ‡
		VI	220	NR	NR ‡	NR ‡
Prashanth et al., 2011 (India) [82]	CS	VI	85	NR	(8–13)	51 (60.0%)
Puteri et al., 2019 (Indonesia) [83]	CS	VI	34	34	(6–16)	20 (58.8%)
Qureshi et al., 2022 (Pakistan) [84]	CS	HI	150	NR	6.4 ± 1.5 (5–8)	67 (44.7%)
Rajab & Mismar, 2023 (Jordan) [85]	CS	VI	161	NR	(6–18)	NR
Reddy & Sharma, 2011 (India) [86]	CS	VI	128	100	(6–15)	73 (57.0%)
Reddy et al., 2013 (India) [87]	CS	HI	95	NR	(7–17)	NR
		VI	48	NR	(7–17)	NR
Rezaei et al., 2019 (Iran) [88]	CS	HI	37	NR	(6–12)	NR ‡
		VI	16	NR	(6–12)	NR ‡
		HI + VI *	2	NR	(6–12)	NR ‡
Samal et al., 2024 (India) [89]	CS	HI	91	NR	15.5 ± 2.5 (5–19)	56 (61.54%)
Samnieng et al., 2014 (Thailand) [90]	CS	VI	146	NR	48.8 ± 5.9	70 (47.9%)
Sandeep et al., 2016 (India) [91]	CS	HI	180	NR	10.8 ± 2.9 (6–16)	93 (51.7%)
Sanjay et al., 2014 (India) [92]	CS	HI	195	NR	(6–20)	NR ‡
		VI	115	NR	(6–20)	NR ‡
Scardina et al., 2007 (Italy) [93]	CS	VI	30	NR	(16–50)	NR
Sharififard et al., 2021 (Iran) [94]	CS	VI	130	NR	14.5 ± 1.6 (12–17)	93 (71.5%)
Sharififard et al., 2022 (Iran) [95]	CS	VI	47	NR	8.9 ± 1.4 (7–11)	29 (61.7%)
Shetty et al., 2010 (India) [96]	CS	VI	221	NR	(6–12)	97 (43.8%)
Shivakumar et al., 2018 (India) [97]	CS	HI	100	NR	(5–18)	NR
Shyama et al., 2001 (Kuwait) [98]	CS	HI	312	NR	13.2	NR ‡
		VI	65	NR	11.4	NR ‡
Singh et al., 2017 (India) [99]	CS	VI	423	NR	12.3 ± 2.3 (9–15)	292 (69.0%)
Singh et al., 2019 (India) [100]	CS	HI	250	NR	12.1 ± 2.5 (9–15)	136 (54.4%)
Sihag et al., 2022 (India) [101]	CS	HI	330	NR	(4–15)	185 (56.1%)
		VI	213	NR	(4–15)	119 (55.9%)
Srivastava et al., 2024 (India) [102]	CS	VI	69	NR	19.7 ± 4.8 (7–28)	55 (79.7%)
Suma et al., 2011 (India) [103]	CS	HI	76	NR	(5–18)	47 (61.8%)
Suresan et al., 2017 (India) [104]	CS	VI	238	NR	(4–23)	139 (58.4%)
Swain et al., 2018 (India) [105]	CS	HI	152	NR	10.2 (5–16)	82 (53.9%)
Tagelsir et al., 2013 (Sudan) [106]	CS	VI	79	NR	11.8 ± 3.1 (6–18)	55 (69.6%)
Tefera et al., 2022a (Ethiopia) [107]	CS	HI	149	NR	15.5 ± 3.9 (7–30)	64 (43.0%)
Tefera et al., 2022b (Ethiopia) [108]	CS	VI	130 ¶	NR	(7–30)	NR ‡
Trimeridou et al., 2024 (Greece) [109]	CS	HI ^a^	33	NR	10.9 ± 2.6 (6.6–15.3)	24 (72.7%)
		HI ^b^	53	NR	16.2 ± 1.3 (13–18)	33 (62.3%)
Tugeman et al., 2016 (Malaysia) [110]	CC	HI	64	69	12.0 ± 2.12 (7–14)	30 (46.9%)
Vichayanrat & Kositpumivate, 2014 (Thailand) [111]	CS	HI	97	83	22.2 ± 2.3	30 (30.9%)
Vishnu et al., 2021 (India) [112]	CS	HI	371	NR	5, 12, 15	NR
	CS	VI	371	NR	5, 12, 15	NR
Vyavahare et al., 2024 (India) [113]	CS	HI	60	80	13.6 ± 1.1 (12–15)	39 (65.0%)
Watson et al., 2010 (UK) [6]	CS	VI	100	NR	NR ◇	45 (45.0%)

CC, case–control; CS, cross-sectional; HI, hearing impairment; M, mean; Nc, number of control participants; NpI, number of participants with impairment; NR, not reported; SD, standard deviation; VI, visual impairment; ^a^ elementary school; ^b^ high school; * not analysed separately; † 100% female; ‡ reported only for the total sample; # median age (IQR) reported instead of M ± SD: HI 13.0 (6.0); VI 12.0 (4.0); § reported sex distribution in the blind group (21 girls, 24 boys) does not correspond to the total sample size reported by the authors (*n* = 50); ¶ data extracted from the visual impairment subgroup; hearing-impaired participants from the same study population were reported separately in Tefera et al. (2022a) [107]; ◇ Participant age not reported; median age at onset of visual impairment was 52 years; 54% of participants were aged ≥75 years.

**Table 4:** Summary of the risk of bias assessment of the included studies.

Risk of Bias Category	Studies [*n*] (%)
Low	18 (21.4)
Moderate	45 (53.6)
High	21 (25.0)
Total	84

**Table 5:** Oral health outcomes in individuals with hearing impairment.

Study (Country)	NpI [*n*]	Oral Hygiene	Periodontal Status	Caries	Other Findings	Treatment Needs
Abd. Rahman et al., 2015 (Malaysia) [37]	63	NR	NR	Primary dentition caries 92.0%; dft 6.1 ± 4.14; Permanent dentition caries 59.0%; DMFT 4.9 ± 3.28	Salivary flow rate 0.14 ± 0.08 mL/min; salivary pH 6.8 ± 0.79	Pit and fissure sealants 83.1%; restorations 83.1%; topical fluoride 64.6%; extractions 44.6%; pulp care 12.3%; no treatment required 3.1%
Al-Maweri et al., 2015 (Yemen) [40]	92	PI 1.19 ± 0.54	GI 1.13 ± 0.60	dmft 4.37 ± 3.11; DMFT 1.91 ± 2.07	NR	NR ‡
Al-Qahtani & Wyne, 2004 (Saudi Arabia) [41]	80	Poor oral hygiene 21.7–47.4%	NR	Caries 93.0–95.7%; dmft 2.11–7.35; DMFT 0.87–5.12	NR	Preventive and restorative dental care recommended
Alkahtani et al., 2019 (Saudi Arabia) [42]	146	OHI-S 1.64; PI 1.35	GI 60.1%	Caries 82.2%; DMFT 10.1 ± 9.8	GOHAI-Ar 14.44 ± 9.59	Treatment needs prevalence 85.6%
Alyami et al., 2022 (Saudi Arabia) [44]	116	NR	Gingivitis 63.79%	DT 21.5%; MT 10.3%; FT 25.8%	Malocclusion: Class II div I 17.24%; Class II div II 9.48%; Class III 4.31%; spacing 14.65%; rotation 8.6%; mild fluorosis 27.58%	Poor oral hygiene status reported; need for improved oral care emphasized
Azfar et al., 2018 (Pakistan) [47]	106	OHI-S 1.45 ± 1.01; good oral hygiene 48.1%; fair 46.2%; poor 5.7%	NR	Caries 68.9%; DMFT 7.58 ± 2.62	57.5% had never visited a dentist; 59.4% brushed once daily; 91.5% consumed confectioneries; 83.0% consumed sweetened juices; 73.6% consumed sweetened milk	Comprehensive dental care programmes and promotion of parental oral health awareness recommended.
Bakardjiev, 2025(Bulgaria) [48]	37	NR *	NR	DMF (T + t) 5.29 ± 0.49 (controls), 6.27 ± 1.03 (mild/moderate HI), 8.31 ± 2.93 (severe HI)	Frequent carbohydrate intake and low socioeconomic status associated with higher DMF; poorer oral hygiene with increasing hearing-loss severity	Targeted preventive interventions recommended
Bimstein et al., 2014 (USA) [49]	85	Generalized plaque deposits 21.9%; heavy deposits 4.9%	Gingival inflammation 80.5%; BOP 18.3%; calculus 25.0%	Caries present 82.0%	Lower dependence on caregivers than VI group	Preventive oral hygiene support recommended
Brozd-Topolko et al., 1988 (Croatia) [50]	83	NR	NR	Caries 87.95%; KEP/DMF 5.08; 12.05% caries-free vs. 21.69% controls	Orthodontic anomalies present in 57.83% of hearing-impaired children; primary compression anomalies most frequent (47.91%).	Organized dental care and preventive supervision emphasized as necessary
Buela et al., 2024 (India) [13]	112	Poor oral hygiene 40.2%	Gingival bleeding 75.0%; periodontal pockets 37.5%	dft 1.82 ± 1.61; DMFT 1.82 ± 1.67	Severe malocclusion 13.4%	Oral prophylaxis + one-surface restoration most common need (42.0%); trauma treatment needed 2.7%
Djeri et al., 2013 (Bosnia & Herzegovina) [57]	66	NR	NR	DMFT 7.79 ± 4.76; DT 6.68 ± 4.45; MT 0.39 ± 0.94; FT 0.71 ± 1.27; DMFT 8.68 (rural) vs. 7.13 (urban); DMFT 9.65 (foster care) vs. 9.50 (institution) vs. 7.10 (parents)	51% brushed twice daily; 6% never brushed teeth; 25.8% had never visited a dentist; 21.2% had not visited a dentist within the previous year	Need for improved access to dental care, oral health education, and caregiver involvement emphasized
Gaçe et al., 2014 (Albania) [59]	147	OHI-S 2.42	NR	Primary dentition caries 65.9%; permanent dentition caries 88.4%; deft 2.8 ± 2.9; DMFT 4.7 ± 3.9	NR	Increased dental treatment needs reported; preventive and restorative dental care recommended.
Ghannam et al., 2024a (Syria) [14]	90	PI 1.5–1.9	GI 0.9–1.1	Caries 93%; DMFT 2.3–2.5; dmft 3.3–4.1	POQL 6.4 ± 2.89; positive correlations between OHRQoL and DMFT (*p* < 0.001), dmft (*p* < 0.001), PI (*p* < 0.001), and GI (*p* = 0.038)	Specialized oral health education and comprehensive dental care recommended
Ghannam et al., 2024b (Syria) [61]	90	PI 2.05 ± 0.80	GI 1.52 ± 0.90	DMFT 2.47 ± 1.86; dmft 4.17 ± 3.12	Higher HI severity associated with increased PI (*p* < 0.001) and GI (*p* < 0.001)	Early multidisciplinary oral and hearing healthcare recommended
Goud et al., 2021 (India) [10]	207	OHI-S 2.43 †	Pockets 4–5 mm 1.5%; calculus 78.5%; gingival bleeding 3.1%; periodontally healthy 16.9%	DMFT 1.24; dmft 0.36.	NR	One-surface fillings most common treatment need (95%); preventive care and fissure sealants needed in 15% of children
Jain et al., 2008 (India) [62]	127	NR	NR	DMFT 2.61; dmft 0.83; DT 83.92%; FT 7.14%.	DMFT increased with age	111/127 (87.4%) required treatment; one-surface fillings 79.5%; two-surface fillings 22%; pulp treatment < 7%; preventive care/fissure sealants needed in 15%
Jain et al., 2013 (India) [63]	297	NR	Shallow pockets 35.4%; calculus 11.1%; bleeding 21.2%; deep pockets 8.1%; periodontally healthy 24.2%	DMFT 1.97 ± 1.93; dft 0.26 ± 0.85	NR	One-surface fillings most common treatment need (60.2%); two-surface fillings 16.2%; pulp care/restoration 10.2%; extraction 12.7%; orthodontic treatment 13.3%
Jnaneswar et al., 2017 (India) [65]	540	NR	Calculus 47.2%; BOP 23.9%; periodontally healthy 28.9%.	Caries 19.3%; DMFT 0.37 ± 0.88; deft 0.15 ± 0.56	NR	Need for preventive care, oral health education, and improved access to dental services emphasized
Kar et al., 2016 (India) [68]	236	NR	NR	Caries 30.51% in HI vs. 15.81 (controls (*p* < 0.05)); 69.49% of HI children caries-free vs. 84.19% (controls)	Higher CAST severity scores in HI children; significantly more pulp involvement, abscess/fistula, and tooth loss due to caries	Need for improved oral health awareness and preventive dental care emphasized
Kim et al., 2024 (South Korea) [70]	1384	NR	NR	NR	Fewer residual natural teeth (19.89 ± 0.43 vs. 25.77 ± 0.18; *p* < 0.001); poorer maxillary and mandibular prosthesis status (*p* < 0.001); poorer chewing function (*p* < 0.001); poorer speaking function (*p* < 0.001)	Regular dental treatment and government-supported oral healthcare programmes recommended
Li et al., 2023 (China) [72]	56	NR	Calculus 42.86%; Gingival bleeding 17.86%.	Caries 66.07%; DMFT 2.57 ± 2.83	Frequency of brushing/day and parental educational background significantly associated with caries experience	Oral health promotion and preventive programmes recommended
Mishra et al., 2021 (India) [75]	277	Fair oral hygiene 41.6%; poor oral hygiene 9.4%	NR	Caries 71.0%; DMFT 1.48 ± 1.61; dmft 0.36 ± 1.04	NR	NR ‡
Mungan Durankaya et al., 2025 (Turkey) [77]	40	Poor oral hygiene 55.0% (CI) vs. 13.6% (NH)	NR	DMFT/dmft 5.48 ± 3.69 (CI) vs. 3.31 ± 2.57 (NH)	Lower maternal education associated with poorer oral health; later initiation and lower frequency of toothbrushing; fewer dental visits in CI children	Need for improved oral hygiene practices and parental awareness
Pradhan et al., 2015 (Nepal) [80]	81	NR	Periodontal index 0.38 ± 0.74 (deaf), 0.19 ± 0.27 (hard of hearing); gingival inflammation 24.7%; destructive periodontal disease 9.6%	DMFT 1.95 ± 3.53 (deaf), 3.16 ± 3.82 (hard of hearing)	56.8% brushed once daily; 34.6% brushed ≥2/day; only one participant used dental floss; 22.2% had never visited a dentist; pain/trouble with teeth and gums was the most common reason for dental visits (50.8%); 22.1% reported chewing difficulties	Need for improved oral health awareness and reduction of communication barriers
Prasad et al., 2018 (India) [81]	460	OHI-S 1.15 ± 0.43; good 64.8%; fair 35.2%	Gingival bleeding 60.7%; pockets 42.0%	Caries 51.7%; DMFT 1.00 ± 1.24	NR	Improved access to dental services and oral health education recommended
Qureshi et al., 2022 (Pakistan) [84]	150	NR	NR	Caries 74.67%	NR	Need for improved oral health awareness and preventive programmes
Reddy et al., 2013 (India) [87]	95	OHI-S 1.15 ± 0.72	NR	DMFT in HI 1.4 ± 1.95; deft 0.47 ± 1.01	None of the children had ever visited a dentist	High unmet dental treatment needs
Rezaei et al., 2019 (Iran) [88]	37	PI 0.86 ± 0.15	GI 1.39 ± 0.30	DMFT 1.81 ± 2.16; dmft 2.08 ± 3.48	Only 27% brushed teeth once daily; no flossing reported	High unmet treatment needs (UTN 0.85 ± 0.31)
Samal et al., 2024 (India) [89]	91	NR	Gingival bleeding 82.4%	Caries 76.9%; DMFT 3.47 ± 3.0	82/91 (90.1%) had never received dental care/visited a dentist; poor perceived oral health; significant association between severity of hearing loss and caries presence (*p* = 0.027)	Considerable treatment needs; 48.4% required prompt treatment and 25.3% urgent treatment
Sandeep et al., 2016 (India) [91]	180	PI 1.70 ± 0.61	Moderate-to-severe gingivitis 78%; GI 1.59 ± 0.58	Caries 65%; DMFT 1.6 ± 1.3 (6–8 y), 1.9 ± 1.2 (9–12 y), 2.2 ± 1.2 (13–16 y)	NR	91.7% required treatment
Sanjay et al., 2014 (India) [92]	195	NR	NR	DMFT in HI 1.80 ± 1.26; dft in HI 0.33 ± 0.24	NR	High unmet dental treatment needs; urgent need for comprehensive dental care
Shivakumar et al., 2018 (India) [97]	100	OHI-S 1.63 ± 0.84; PI 0.85 ± 1.29	NR	DMFT/dmft 2.74 ± 1.58; DT 2.83 ± 0.94; untreated caries 81.2%	Higher caries experience in older age groups	Extensive unmet dental treatment needs; preventive and curative dental care emphasized
Shyama et al., 2001 (Kuwait) [98]	312	Good oral hygiene 11.7%; poor 88.3%	NR	Caries: dmft 5.3; DT 4.3; DMFT 5.0; DMFS 9.0	NR	Strengthened organized preventive and restorative dental care recommended
Singh et al., 2019 (India) [100]	250	NR	NR	Caries 56%; DMFT 1.66 ± 2.09	TDI 40.8%; crowding 49.6%; less favorable OHRQoL	Need for improved oral health promotion and preventive care
Sihag et al., 2022 (India) [101]	330	Fair oral hygiene 40.0%; poor 30.0%	NR	Caries prevalence 48.5%; DMFT 1.2 ± 1.1; dmft 0.4 ± 0.2	NR	School-based oral health education, regular dental check-ups and prompt treatment recommended
Suma et al., 2011 (India) [103]	76	NR	Gingivitis 35%	Caries 42%	Toothbrushing once daily 82.9%; 80.3% had never visited a dentist; malocclusion 19%; fractured anterior teeth 3.9%	87% required single- or double-surface restorations; remaining children required pulp therapy
Swain et al., 2018 (India) [105]	152	Poor oral hygiene 42%	Gingivitis 15.8%; gingivitis score 1.70 ± 0.61.	Caries 45.39%; DT 3.2 ± 2.1; MT 0.9 ± 1.2; FT 0.4 ± 1.2	Toothbrushing once daily 82.9%; 80.3% had never visited a dentist; malocclusion 19%; fractured anterior teeth 3.9%; lack of awareness among caregivers 21.1%	Preventive oral health programme and caregiver education recommended
Tefera et al., 2022a (Ethiopia) [107]	149	Poor oral hygiene 31.5%	Periodontal disease 22.8%; BOP 43.6%	Caries 38.9%; DMFT 1.15 ± 1.65; dmft 0.11 ± 0.56	NR	School oral health programmes and caregiver-assisted oral hygiene practices recommended
Trimeridou et al., 2025 ^a^ (Greece) [109]	33	NR	NR	dmfs 2.5 ± 5.2; dmft 1.0 ± 1.9; DMFS 3.5 ± 5.4; DMFT 1.9 ± 2.4; 30.3% caries-free	NR	NR
Trimeridou et al., 2025 ^b^ (Greece) [109]	53	NR	NR	DMFS 7.3 ± 9.0; DMFT 3.8 ± 3.9; 24.5% caries-free	NR	NR
Tugeman et al., 2016 (Malaysia) [110]	64	Mature plaque 50.8%; acid-producing plaque 14.8%; DPMS 1.8 ± 0.57; significantly higher dental plaque maturity scores than controls (*p* < 0.001)	NR	NR	Poor oral health knowledge and oral health practices compared with controls; brushing before bedtime 46.4%; regular dental visits 62.9%	Alternative oral health education tailored to HI children recommended
Vichayanrat et al., 2014 (Thailand) [111]	97		NR	Caries 82.5%; DMFT 3.90 ± 3.22	HI students had fewer filled teeth and fewer missing teeth than controls	Oral health education recommended
Vishnu et al., 2021 (India) [112]	371	Poor oral hygiene 58.0%	Gingival bleeding 63.1%	Caries 34.2%	NR	High unmet treatment needs; one-surface restoration 18.9%; two-surface restoration 14.6%; pulp therapy 11.1%
Vyavahare et al., 2024 (India) [113]	60	NR	NR	Caries 83.3% vs. 51.2% (controls); DMFT 6.80 ± 6.00 vs. 1.26 ± 1.72 (controls)	70% classified as very high caries risk; reduced salivary flow and buffering capacity associated with caries risk	Tailored preventive and curative dental programmes recommended

BOP, bleeding on probing; CAST, Caries Assessment Spectrum and Treatment; CI, cochlear implant; deft, decayed, extracted, and filled primary teeth; dft, decayed and filled primary teeth; DMF (T + t), caries experience index used in mixed dentition; dmft/DMFT, decayed, missing, and filled primary/permanent teeth; DMFS, decayed, missing, and filled surfaces; DPMS, dental plaque maturity score; DT, decayed teeth; FT, filled teeth; GI, gingival index; GOHAI-Ar, Geriatric Oral Health Assessment Index, Arabic version; HI, hearing impairment; HLG, hearing loss group; KEP, decayed, extracted and filled teeth index; MT, missing teeth; NH, normal hearing; NHLG, normal hearing group; NpI, number of participants with impairment; NR, not reported; OHI-S, Oral Hygiene Index, Simplified; OHRQoL, oral health-related quality of life; PI, plaque index; POQL, Pediatric Oral Quality of Life Questionnaire; POHRQoL, Pediatric Oral Health-Related Quality of Life; TDI, traumatic dental injuries; UTN, unmet treatment need; VI, visual impairment; ^a^ elementary school; ^b^ high school; * Oral hygiene was assessed using the Green–Vermillion OHI-S; results were reported according to oral hygiene categories and degree of hearing loss rather than as separate oral hygiene outcomes for the hearing-impaired cohort; † Narrative text reports reversed group values; values presented here are reported according to Table 3; ‡ Treatment needs were reported only for the overall study population; no subgroup-specific data were available.

**Table 6:** Oral health outcomes in individuals with visual impairment.

Study (Country)	NpI [*n*]	Oral Hygiene	Periodontal Status	Caries	Other Findings	Treatment Needs
Ajeel & Diab, 2024 (Iraq) [39]	70	NR	NR	DMFT 2.30 vs. 1.20; DMFS 3.03 vs. 1.53; dmft 5.26 vs. 5.03; dmfs 15.76 vs. 13.59	Decreased salivary flow rate; increased salivary TGF-β1	Early oral health promotion and preventive programmes recommended
Al-Maweri et al., 2015 (Yemen) [40]	50	PI 1.62 ± 0.54	GI 1.84 ± 0.79	dmft 3.44 ± 3.10; DMFT 1.44 ± 1.56	NR	NR §
Al-Qahtani & Wyne, 2004 (Saudi Arabia) [41]	29	Poor oral hygiene 16.7–17.6%	NR	Caries 88.2–100%; dmft 1.00–7.58; DMFT 1.67–3.80	NR	Preventive and restorative dental care recommended
AlSadhan et al., 2017 (Saudi Arabia) [43]	79	OHI 2.06 ± 0.84 vs. 1.31 ± 0.53 (*p* = 0.001); PI 1.72 ± 0.59 vs. 1.32 ± 0.52 (*p* < 0.0001)	GI 1.35 ± 0.56 vs. 1.06 ± 0.25 (*p* = 0.001)	DMFS 5.16 ± 8.1 vs. 3.10 ± 3.7 (*p* = 0.03)	Medical conditions more common in VI (21.5% vs. 4.8%); most frequent psychological problems (5.1%), diabetes (3.8%), and epilepsy (3.8%)	Oral health educational, preventive and curative programmes recommended
Amrollahi et al., 2020 (Iran) [45]	50	NR	NR	DMFT 2.40 ± 1.32	Fewer missing teeth and more filled teeth associated with higher parental awareness	Need for parental oral health education and preventive care
Anand et al., 2021 (India) [46]	180	OHAT oral cleanliness: changes 81.1%; unhealthy 5.0%	OHAT gingival changes 54.4%; unhealthy 8.9%	56.1% had 1–3 decayed/broken teeth; 13.9% had ≥4 decayed/broken teeth	Altered salivary status 40%; dental pain signs 25.6%; halitosis reported	Considerable oral treatment needs indicated by OHAT findings
Bimstein et al., 2014 (USA) [49]	35	Generalized plaque deposits 45.7%; heavy deposits 17.4%	Gingival inflammation 85.7%; bleeding on probing 42.9%; calculus 48.6%	Caries present 71.0%	Greater dependence on caregivers than HI group (*p* < 0.001)	Enhanced preventive oral hygiene support recommended
Brozd-Topolko et al., 1988 (Croatia) [50]	83	NR	NR	Caries 92.77%; KEP/DMFT 5.76	Orthodontic anomalies present in 69.87% (24.69% higher than in healthy controls); primary compression anomalies in 25.86%	Need for intensified preventive dental care and early orthodontic assessment
Buela et al., 2024 (India) [13]	78	Poor oral hygiene 42.3%	Gingival bleeding 82.1%; periodontal pockets 42.3%	dft 2.29 ± 1.71; DMFT 2.38 ± 1.86	Severe malocclusion 37.2%	Oral prophylaxis + one-surface restoration 35.9%; trauma treatment 11.5%
Cunha & Bizarra, 2023 (Portugal) [51]	34	OHI-S 0.61 ± 0.40 (VI); 0.48 ± 0.32 (sighted)	GI 0.18 ± 0.25 (VI); 0.13 ± 0.19 (sighted)	dmft 1.60 ± 2.93 vs. 0.83 ± 2.60; DMFT 0.29 ± 0.68 vs. 0.35 ± 0.77	VI children required brushing assistance more frequently (85.7%) and attended dental check-ups less regularly (69%)	Oral health education recommended for children with VI and their parents
da Cunha et al., 2015 (Brazil) [52]	52	Interproximal PI 66.8 ± 28.3 (mild/moderate VI); 43.2 ± 23.3 (severe/profound VI); 32.7 ± 31.1 (blind)	Interproximal CAL 1.88 vs. 1.42; total PD 1.88 vs. 1.62; interproximal PD 1.86 vs. 1.43 (acquired vs. congenital VI)	NR	Poor knowledge of fluoride (67.3%), dental biofilm (96.2%), and calculus (94.2%)	Preventive oral hygiene education and dental care recommended
Darwita et al., 2024 (Indonesia) [53]	25	Good DI-S (72%); fair DI-S (28%)	NR	Caries 64%; dmft 2.24 ± 3.24; DMFT 0.96 ± 1.02	Maxillary crowding 44%; mandibular crowding 48%	40% required dental treatment (20% immediate; 20% non-immediate)
Daryani et al., 2014 (India) [54]	60	High plaque score: 25.0% (VI) vs. 15.0% (controls); moderate plaque score: 31.7% vs. 30.0%	NR	NR	Reduced salivary buffer capacity 53.3%; reduced salivary flow rate 33.3%; lower predicted chance of avoiding future caries 41% vs. 74% (controls); salivary and microbial factors associated with caries risk	Intensive preventive strategies and caries risk assessment recommended
Debnath et al., 2025 (India) [55]	260	Fair oral hygiene 43.1%; poor 0.4%	Gingival bleeding 52.3%	DT 0.67; MT 0.03; FT 0.01; DMFT = 0.71	NR	Increased oral health awareness and improved accessibility of oral healthcare recommended
Dhanvanth et al., 2020 (India) [56]	20	OHI 2.3	Generalized chronic gingivitis 55%; localized periodontitis 30%; generalized chronic periodontitis 15%	NR	NR	Restorations 85%; prophylaxis 70%; extractions 45%; prostheses 15%
Dogra et al., 2019 (India) [58]	1000	NR	NR	Caries 39.83% (CAST); 36.58% (ICDAS-II)	Incipient white spot lesions: 54.57% (CAST); 48.07% (ICDAS-II code 1); 53.06% (ICDAS-II code 2)	Early remineralization and preventive interventions recommended
Dumančić et al., 2025 (Croatia) [9]	85	NR	NR	DMFT 17.0 (IQR 12.5–22.0)	Toothbrushing ≥ 2× daily in 72.9%; dental floss use 28.2%; higher D-component associated with smoking and soft drink consumption	Organized preventive care and improved accessibility of dental services recommended
Feng et al., 2025 (China) [16]	101	NR	NR	Caries 92.1%; dmft 5.75; DMFT 5.80	Toothbrushing ≥ 2× daily in 52.1%; independent brushing 84%; fluoride toothpaste use 39.4%; altered salivary microbiota; Actinomycetota associated with gingivitis and caries	School-based oral health promotion and preventive programmes recommended
Gaçe et al., 2014 (Albania) [59]	91	OHI-S 1.62	NR	Primary dentition caries 81.9%; permanent dentition caries 91.0%; deft 4.4 ± 4.2; DMFT 4.7 ± 3.9	NR	Increased dental treatment needs reported; preventive and restorative dental care recommended
Gaikwad et al., 2022 (India) [60]	400	Poor oral hygiene 25%	NR	Caries 90.3%; DMFT 4.5 ± 2.7; DT 3.1; MT 0.8; FT 0.5	Oral malodor 48%; tooth sensitivity 50%; limited use of mouthwash and floss	Oral health promotion programmes and practical oral hygiene education recommended
Goud et al., 2021 (India) [10]	77	OHI-S 1.95 *	8.8% gingival bleeding; 86.8% calculus †	DMFT 0.98; dmft 0.52	Dental fluorosis 5%; tongue thrusting, mouth breathing, and thumb sucking reported	One-surface fillings most common treatment need (95%); fissure sealants needed in 15% of children
Hazra et al., 2022 (India) [15]	50	Poor oral hygiene 38%; significantly poorer oral hygiene than controls (*p* < 0.001)	Moderate gingivitis 80%; severe 18%; significantly poorer gingival health than controls (*p* < 0.001)	deft 0.92 ± 1.75; DMFT 2.44 ± 1.79; carious lesions 3.36 ± 2.59	Lower salivary TAC levels in VI children (49.17 ± 25.70 vs. 156.67 ± 56.03 μg/mL; *p* < 0.001); inverse correlation between TAC and caries, oral hygiene, and gingival status	Antioxidant-rich diet and reinforced oral care efforts recommended
Jain et al., 2013 (India) [63]	201	NR	Gingival bleeding 8.9%; calculus 13.4%; shallow pockets 28.4%; deep pockets 6.0%	DMFT 1.48 ± 1.29; dft 0.28 ± 0.75	Better periodontal health than HI subjects (43.3% vs. 24.2% periodontally healthy)	One-surface fillings 55.2%; two-surface fillings 9.0%; pulp care/restoration 19.4%; extractions 22.3%
Jawahar & Maragathavalli, 2022 (India) [64]	207	NR	Gingival bleeding 94.69%; periodontal pockets 81.16%; attachment loss 58.9%	Caries 62.3%; DT 62.3%; MT 60.3%; FT 9.7%	Oral mucosal lesions 14%; dental trauma 30%; tobacco habits 16.4%	Prompt dental treatment required in 71%; immediate treatment in 28%; no treatment needed in 1%
John et al., 2017 (India) [66]	404	Poor oral hygiene 25%	NR	Caries 90.3%; DMFT 4.5 ± 2.7; DT 3.1 ± 2.2	Toothbrushing once daily 69%; oral malodour 48.1%; tooth sensitivity 48%; mouthwash use 21.8%; previous dental visit 54.7%	Comprehensive dental care, oral health education and regular screening recommended
Kannappan et al., 2023 (India) [67]	130	Poor oral hygiene 0%	NR	Permanent dentition caries 40%; primary dentition caries 63.1%; DMFT 0.57 ± 0.80; deft 1.13 ± 1.11	TDI 35.4%	Primary prevention and oral health education for parents and teachers recommended
Khan et al., 2020 (Malaysia) [69]	91	OHI-S 1.68 ± 0.87; good oral hygiene 30.8%; fair 39.6%; poor/very poor 29.7%	NR	DMFT 0.80 ± 1.62	NR	Regular oral health education and oral examinations recommended
Lee et al., 2024 (Hong Kong) [71]	73	VPI 0.76 ± 0.30	NR	Caries 43.8%; DMFT 1.0 ± 1.8; D-component 80% of DMFT	Toothbrushing ≥ 2× daily 90.4%; interdental cleaning 20.5%; difficulties accessing dental care reported by 43.8% of caregivers	Preventive oral health measures for visually impaired students and caregivers recommended
Li et al., 2023 (China) [72]	118	NR	Gingival bleeding 52.08%; calculus 59.38%	Caries 66.10%; DMFT 2.71 ± 3.06; first permanent molar caries 52.54%	49.16% had no fixed brushing method; 13.6% brushed <1× daily or never; low parental education associated with higher DMFT; fluoride use associated with lower caries experience	Comprehensive oral health interventions and health promotion recommended
Liu et al., 2019 (China) [73]	103	NR	Gingival bleeding 44.66%; calculus 67.96%	Caries 78.64%; DMFT 2.43 ± 2.75	Malocclusion 49.51%; first permanent molar caries higher in blind than low-vision children	Improved oral health education, parental education, and fluoride-based caries prevention recommended
Macharia et al., 2023 (Kenya) [74]	159	Plaque score 0.95 ± 0.45	Gingivitis 88.1%; GI 0.28 ± 0.25	Caries 44.7%; DMFT 0.44 ± 0.60; dmft 0.12 ± 0.32	Toothbrushing ≥ 2× daily in 67.3%; 93.1% unaware whether toothpaste contained fluoride; toothbrush replacement frequency associated with gingival status (*p* < 0.001)	Sustainable oral health promotion programmes recommended
Mishra et al., 2021 (India) [75]	97	Fair oral hygiene 56.7%; poor 12.4%	NR	Caries prevalence 71.0%; mean DMFT 1.48 ± 1.61; mean dmft 0.36 ± 1.04	NR	NR §
Ozdemir-Ozenen et al., 2012 (Turkey) [78]	50	PI 1.50 ± 0.18; OHI-S 1.72 ± 0.21	GI 0.68 ± 0.12	DMFT 1.62 ± 0.20; DMFS 1.87 ± 0.80; dft 4.35 ± 1.50; dfs 5.85 ± 1.20	NR	Oral health education, dietary guidance, and regular dental follow-up recommended
Parkar et al., 2014 (India) [79]	160	Moderate oral hygiene; OHI-S increased significantly with age (*p* = 0.003)	NR	Caries 46.9%; dmft 0.37 ± 0.87; DMFT 1.14 ± 1.74	NR	68.1% required treatment; one-surface fillings 67.9%; two-surface fillings 13.8%; crowns 7.3%; pulp care/restoration 6.4%; extractions 4.6%
Prasad et al., 2018 (India) [81]	220	OHI-S 1.70 ± 0.63; good 22.7%; fair 73.7%; poor 3.6%	Gingival bleeding 89.5%; pockets 43.1%	Caries 63.2%; DMFT 1.32 ± 1.24	Anterior maxillary/mandibular overjet or open bite 10.9% (*p* < 0.001)	Improved access to dental services and oral health education recommended
Prashanth et al., 2011 (India) [82]	85	OHI-S 0.58	NR	DMF 0.6; def 2.0; primary dentition caries 69.4%; permanent dentition caries 35.2%	Awareness of sugar-caries relationship 51.8%; caregiver support considered important	Parental/caregiver support, oral health education, and diet counseling recommended
Puteri et al., 2019 (Indonesia) [83]	34	NR	NR	Caries index 1.5	Good oral health knowledge 82.4%; positive attitude 67.6%; good oral health practice 58.8%; oral health knowledge associated with CI (*p* = 0.03)	Community-based oral health promotion and caries prevention programmes recommended
Rajab & Mismar, 2023 (Jordan) [85]	161	NR	Gingival bleeding 84%	Caries 73.3%; DMFT/dmft 3.86; untreated decay predominant; 6–<12 y: 86.4% (dmft 5.17); 12–18 y: 65.7% (DMFT 2.68)	Toothbrushing reported in 80.8%; <33% brushed ≥1× daily; fluoridated toothpaste use ≈ 50%; TDI 19.3%; higher in complete VI (28.5% vs. 15.5%); dental aesthetic concerns 45.3%; bullying due to dental problems 7.5%	Tailored oral health education, regular dental visits, fluoridated toothpaste use, dietary counselling and preventive interventions recommended
Reddy & Sharma, 2011 (India) [86]	128	Fair oral hygiene 22%; poor 40%	NR	Caries 40%; DMFT/deft 1.1/0.17	Higher TDI than controls (0.29 vs. 0.13)	Need for comprehensive dental care and preventive oral health measures emphasized
Reddy et al., 2013 (India) [87]	48	OHI-S 1.51 ± 0.93	NR	DMFT 0.94 ± 1.45; deft 0.19 ± 0.79	None had ever visited a dentist; untreated dental pain reported	Oral health education and guardian training recommended
Rezaei et al., 2019 (Iran) [88]	16	PI 0.86 ± 0.15	GI 1.39 ± 0.30	DMFT 1.31 ± 1.20; dmft 2.81 ± 2.81	Low brushing frequency; none flossed	High unmet treatment needs (UTN 0.76 ± 0.34)
Samnieng et al., 2014 (Thailand) [90]	146	OHI-S 2.52	Periodontal disease 34.8%	DMFT 16.0 (DT 4.4; MT 10.2; FT 1.4)	Chewing problems 45.2%; swallowing problems 32.6%; tasting problems 29.2%; speaking problems 26.5%	35% needed fillings; 12.3% extractions; 38.2% needed upper and lower prostheses; need to develop appropriate education and service delivery programmes to improve the oral health conditions
Sanjay et al., 2014 (India) [92]	115	NR	NR	DMFT 2.16 ± 2.00; dft 0.25 ± 0.27	DMFT increased significantly with age (*p* ≤ 0.001); Higher DMFT in blind than hearing-impaired children (2.16 vs. 1.80)	Comprehensive and incremental dental care recommended
Scardina et al., 2007 (Italy) [93]	30	OHI-S 2.76–4.60	NR	DMFT 5.9–10.0	56.7% had never visited a dentist; poor oral hygiene practices reported	Oral hygiene instruction and preventive oral health promotion recommended
Sharififard et al., 2021 (Iran) [94]	130	OHI-S 2.01 ± 0.70; OHI-S > 1.8 in 60%	BOP 79.2%	DMFT 2.43 ± 2.24; caries 72.3%; DMFT ≥ 3 in 45.4%	13.8% had never visited a dentist; only 30.8% had a dental visit in the previous 12 months	High burden of untreated caries (62.3% with ≥1 decayed tooth)
Sharififard et al., 2022 (Iran) [95]	47	OHI-S 2.09 ± 0.58	BOP 78.7%	dmft 2.85 ± 3.21; DMFT 0.81 ± 1.15; caries-free 23.4%	17.0% had never visited a dentist; 25.5% reported toothache in previous 12 months	Unrestored caries in 57.4% of primary teeth and 34.0% of permanent teeth
Shetty et al., 2010 (India) [96]	221	Fair 51.6%; poor 39.4%	Moderate gingivitis 55.6%; severe 34.4%	Caries 98.5%; dft 7.26; DMFT 4.87	High proportion of untreated decay	Unmet preventive and restorative treatment needs emphasized
Shyama et al., 2001 (Kuwait) [98]	65	Good oral hygiene 10.3%; poor 89.7%	NR	Caries: dmft 3.7; DT 2.2; DMFT 2.8; DMFS 4.8	NR	Strengthened organized preventive and restorative dental care recommended
Singh et al., 2017 (India) [99]	423	NR	NR	Caries 57.7%; DMFT 1.64; deft 1.53	TDI 50.6%; crowding 61.5%; unfavorable OHRQoL	Specialized oral healthcare programmes and preventive measures recommended
Sihag et al., 2022 (India) [101]	213	Fair oral hygiene 46.5%; poor 29.1%	NR	Caries 45.5%; DMFT 0.8 ± 1.3; dmft 0.6 ± 0.2	NR	School-based oral health education, regular dental check-ups and prompt treatment recommended
Srivastava et al., 2024 (India) [102]	69	NR	BOP 60.9%	Caries 63.8%; DMFT 1.43 ± 1.61	TDI 23.2%; higher in acquired vs. congenital blindness (37.5% vs. 15.6%; *p* = 0.04)	Improved access to oral healthcare and regular dental examinations recommended
Suresan et al., 2017 (India) [104]	238	OHI-S 2.43 ± 1.03	NR	Caries 15% (dmft) and 46% (DMFT); dmft 0.48 ± 1.54; DMFT 1.57 ± 2.30	TDI 4.62% (11/238) ‡; enamel fracture most common; higher prevalence in completely blind children	One-surface fillings 47%; two-surface fillings 21%; extractions 16%; pulp care 13%; high unmet dental treatment needs emphasized
Tagelsir et al., 2013 (Sudan) [106]	79	OHI-S 1.3 ± 0.9	NR	Caries 46.8%; DMFT 0.4 ± 0.7; dmft 1.9 ± 2.8	TDI 19%; Child-OIDP impact in 29% of 11–13-year-olds; PVI children had higher caries risk; CVI children had higher TDI risk	Urgent treatment need in 25.3%; >90% due to deep caries/pulp involvement
Tefera et al., 2022b (Ethiopia) [108]	130 ¶	Poor oral hygiene 43.8%	BOP 56.7%; periodontal disease 30.0%	NR	Higher periodontal disease in VI than HI students (30.0% vs. 22.8%)	Oral health education and regular dental visits recommended
Vishnu et al., 2021 (India) [112]	371	Poor oral hygiene > 70%	Gingival bleeding 78.7%	Caries 42.0%	TDI 8.4%	High unmet treatment needs; one-surface restoration 23.7%; two-surface restoration 16.7%; pulp therapy 15.1%
Watson et al., 2010 (United Kingdom) [6]	100	Visible plaque 59%	Visible calculus 56%	DT 0.9	24% were not registered with a dentist; 26% requested oral health information in an accessible format	Improved access to oral health information in accessible formats recommended

BOP, bleeding on probing; CAL, clinical attachment loss; CAST, Caries Assessment Spectrum and Treatment; Child-OIDP, Child Oral Impacts on Daily Performance; CPI, Community Periodontal Index; CVI, complete visual impairment; deft, decayed, extracted, and filled primary teeth; dft, decayed and filled primary teeth; dmft/DMFT, decayed, missing, and filled primary/permanent teeth; dfs/DMFS, decayed, missing, and filled surfaces (primary/permanent dentition); DT, decayed teeth; FT, filled teeth; GI, gingival index; HI, hearing impairment; ICDAS-II, International Caries Detection and Assessment System II; IQR, interquartile range; MT, missing teeth; NpI, number of participants with impairment; NR, not reported; OHI-S, Oral Hygiene Index, Simplified; OHAT, Oral Health Assessment Tool; OHRQoL, oral health-related quality of life; PD, probing depth; PI, plaque index; PVI, partial visual impairment; RCT, root canal treatment; SD, standard deviation; TAC, total antioxidant capacity; TDI, traumatic dental injury/injuries; UTN, unmet treatment need; VI, visual impairment; VPI, visible plaque index; * Narrative text reports reversed group values; values presented here are reported according to Table 3; † Periodontal status assessed among 68 visually impaired children eligible for CPI examination; ‡ Discrepancy noted: the abstract reports TDI prevalence as 11%, whereas the *Traumatic dental injuries* part of the Results section reports 11/238 participants (4.62%); the value from the *Traumatic dental injuries* part of the Results section was used; § Treatment needs were reported only for the overall study population; no subgroup-specific data were available; ¶ Data extracted from the visual impairment subgroup; hearing-impaired participants from the same study population were reported separately in Tefera et al. (2022a) [107].

**Table 7:** Oral health outcomes in studies including participants with hearing and visual impairments without separate subgroup analyses.

Study	NpI [*n*]	Oral Hygiene	Periodontal Status	Caries	Other Findings	Dental Needs
Agbor et al., 2020 [38]	180	Poor oral hygiene 56%	NR	Caries 77.3%; MT 44%; FT 11%	60% had never visited a dentist; oral health problems affected eating (45%); sleep (22.8%); studying (8.3%).	Scaling/polishing 33%; fillings 33%; extraction 18%; RCT 8%; orthodontic treatment 8%

FT, filled teeth; MT, missing teeth; NpI, number of participants with impairment; NR, not reported; RCT, root canal treatment.

**Table 8:** (**a**) GRADE evidence profile for the primary oral health outcomes in individuals with hearing impairment. (**b**) GRADE evidence profile for the primary oral health outcomes in individuals with visual impairment.

(**a**)							
**Outcome**	**Studies [*n*]**	**Study Design [*n*]**	**Risk of Bias**	**Inconsistency**	**Indirectness**	**Imprecision**	**Certainty of Evidence**
Dental caries	42	40 CS 2 CC	Serious	Serious	Not serious	Not serious	Very low
Oral hygiene	24	23 CS 1 CC	Serious	Serious	Not serious	Serious	Very low
Periodontal status	20	20 CS	Serious	Serious	Not serious	Serious	Very low
Treatment needs *	11	11 CS	Serious	Serious	Not serious	Serious	Very low
(**b**)							
**Outcome**	**Studies [*n*]**	**Study design [*n*]**	**Risk of bias**	**Inconsistency**	**Indirectness**	**Imprecision**	**Certainty of evidence**
Dental caries	53	51 CS 1 CC	Serious	Serious	Not serious	Not serious	Very low
Oral hygiene	30	29 CS 1 CC	Serious	Serious	Not serious	Serious	Very low
Periodontal status	22	21 CS 1 CC	Serious	Serious	Not serious	Serious	Very low
Treatment needs *	20	20 CS	Serious	Serious	Not serious	Serious	Very low

Abbreviations: CC, case–control study; CS, cross-sectional study; GRADE, Grading of Recommendations Assessment, Development and Evaluation; * Only studies reporting quantitative treatment needs were included in the GRADE assessment. Narrative recommendations were not considered.

## Data Availability

No new data were created or analyzed in this study. Data sharing is not applicable to this article.

## Supplementary Materials

The following supporting information can be downloaded at https://www.mdpi.com/article/10.3390/jcm15155765/s1, Supplementary File S1. PRISMA 2020 Checklist [18]; Supplementary File S2. Complete search strategies for Ovid MEDLINE, Ovid Embase, and Web of Science.

## Abbreviations

The following abbreviations are used in this manuscript:

AED	Amsterdam Efficient Deduplication
BOP	Bleeding on probing
Child-OIDP	Child Oral Impacts on Daily Performance
DMF	Decayed, missing, and filled teeth
DMFS	Decayed, missing, and filled surfaces
DMFT	Decayed, missing, and filled teeth
dmft	Decayed, missing, and filled primary teeth
Emtree	Embase thesaurus
GI	Gingival index
GRADE	Grading of Recommendations Assessment, Development and Evaluation;
HI	Hearing impairment
JBI	Joanna Briggs Institute
MeSH	Medical Subject Headings
OHI	Oral Hygiene Index
OHI-S	Oral Hygiene Index—Simplified
OHRQoL	Oral health-related quality of life
PI	Plaque index
PRISMA	Preferred Reporting Items for Systematic Reviews and Meta-Analyses
TDI	Traumatic dental injury/injuries
UTN	Unmet treatment need
TAC	Total antioxidant capacity
VI	Visual impairment
WHO	World Health Organization
