# Supplementary material for: Oral Health Status and Dental Needs in Individuals with Sensory Impairments: A Systematic Review
Source: J Clin Med. 2026 Jul 23;15(15):5765. doi: 10.3390/jcm15155765 (PMC13467371; doi:10.3390/jcm15155765)
Supplement: Supplementary file 1 [file jcm-15-05765-s001.zip › jcm-4430195-File S2.pdf]

Supplementary material

Ovid Medline Search Results – 08 Jan 2026

Search	Query	Results
#5	1 and (2 or 3 or 4)	1,301
#4	(("Persons with Disabilities"/ or "Dental Care for Persons with Disabilities"/ or "Health Services for Persons with Disabilities"/) and ("hearing" or "speech" or "deaf" or "auditory" or "visual" or "blind" or "sensory" or "special school*").ab,ti,kf.) or (((("disab*" or "special need*") adj3 ("person*" or "patient*" or "individual*" or "people" or "dental care")) or "special care dentistry") adj5 ("hearing" or "speech" or "deaf" or "auditory" or "visual" or "blind" or "sensory" or "special school*")).ab,ti,kf.	4,270
#3	"Persons with Visual Disabilities"/ or exp "Vision Disorders"/ or ((("visual*" or "vision*" or "sensory") adj3 ("impair*" or "disorder*" or "disab*" or "loss*" or "defect*" or "damage" or "handicap*" or "function*" or "dysfunction*")) or "impaired vis*" or "blindness*" or "low vision*" or "reduced vision*" or "subnormal vision*" or "abnormal vision*" or "diminished vision*" or "blind indiv*" or "blind people" or "blind person*" or "blind worker*" or "the blind" or "loss of sight" or "sighted" or "poor sight" or "low sight" or "poor vision*" or "reduced sight*").ab,ti,kf.	227,628
#2	exp "Hearing disorders"/ or "Persons with Hearing Disabilities"/ or exp "Hearing Aids"/ or exp "Correction of Hearing Impairment"/ or ((exp "Hearing"/) and ("impair*" or "disorder*" or "disab*" or "loss*" or "defect*" or "damage" or "handicap*" or "function*" or "dysfunction*").ab,ti,kf.) or (("hearing" or "speech" or "sensory") adj3 ("impair*" or "disorder*" or "disab*" or "loss*" or "defect*" or "damage" or "handicap*" or "function*" or "dysfunction*")).ab,ti,kf. or ("hard-of-hearing" or "deaf*" or "auditory rehab*" or "auditory disab*" or "auditory impair*" or "auditory handicap*" or "hypoacus*" or "hearing aid*" or "hearing implant*" or "hearing prosthes*" or "hearing device*" or "cochlear implant*" or "cochlear prosthes*" or "cochlear device*" or ("auditory" and ("implant*" or "prosthes*")) or "poor hearing"	211,667

Search	Query	Results
	or "reduced hearing" or "subnormal hearing" or "abnormal hearing" or "diminished hearing").ab,ti,kf.	
#1	exp "Periodontal Diseases"/ or exp "Dental Caries"/ or exp "Oral Hygiene"/ or "Oral Health"/ or ((("oral" or "dental" OR "gingiv*" or "gum*" or "periodont*") adj3 ("hygien*" or "health" or "status" or "care")) or "tooth loss" or "edentul*" or "missing teeth" or "caries" or "carious").ab,ti,kf.	259,449

Ovid Embase Search Results – 08 Jan 2026

Search	Query	Results
#6	5 not (conference abstract/ or conference review/)	3,330
#5	1 and (2 or 3 or 4)	3,581
#4	((disabled person/ OR physically disabled person/) AND ("hearing" OR "speech" OR "deaf" OR "auditory" OR "visual" OR "blind" OR "sensory" OR "special school*").ab,ti,kf.) OR (((("disab*" OR "special need*") adj3 ("person*" OR "patient*" OR "individual*" OR "people" OR "dental care")) OR "special care dentistry") adj5 ("hearing" OR "speech" OR "deaf" OR "auditory" OR "visual" OR "blind" OR "sensory" OR "special school*")).ab,ti,kf.	3,883
#3	exp visually impaired person/ OR exp visual disorder/ OR ((("visual*" OR "vision*" OR "sensory") adj3 ("impair*" OR "disorder*" OR "disab*" OR "loss*" OR "defect*" OR "damage" OR "handicap*" OR "function*" OR "dysfunction*")) OR "impaired vis*" OR "blindness*" OR "low vision*" OR "reduced vision*" OR "subnormal vision*" OR "abnormal vision*" OR "diminished vision*" OR "blind indiv*" OR "blind people" OR "blind person*" OR "blind worker*" OR "the blind" OR "loss of sight" OR	488,086

Search	Query	Results
	"sighted" OR "poor sight" OR "low sight" OR "poor vision*" OR "reduced sight*").ab,ti,kf.	
#2	exp hearing disorder/ OR exp hearing impaired person/ OR exp hearing aid/ OR exp auditory rehabilitation/ OR ((exp hearing/) AND ("impair*" OR "disorder*" OR "disab*" OR "loss*" OR "defect*" OR "damage" OR "handicap*" OR "function*" OR "dysfunction*").ab,ti,kf.) OR (("hearing" OR "speech" OR "sensory") adj3 ("impair*" OR "disorder*" OR "disab*" OR "loss*" OR "defect*" OR "damage" OR "handicap*" OR "function*" OR "dysfunction*")).ab,ti,kf. OR ("hard-of-hearing" OR "deaf*" OR "auditory rehab*" OR "auditory disab*" OR "auditory impair*" OR "auditory handicap*" OR "hypoacus*" OR "hearing aid*" OR "hearing implant*" OR "hearing prosthes*" OR "hearing device*" OR "cochlear implant*" OR "cochlear prosthes*" OR "cochlear device*" OR ("auditory" AND ("implant*" OR "prosthes*")) OR "poor hearing" OR "reduced hearing" OR "subnormal hearing" OR "abnormal hearing" OR "diminished hearing").ab,ti,kf.	321,815
#1	exp periodontal disease/ OR exp dental caries/ OR exp mouth hygiene/ OR ((("oral" OR "dental" OR "gingiv*" OR "gum*" OR "periodont*") adj3 ("hygien*" OR "health" OR "status" OR "care")) OR "tooth loss" OR "edentul*" OR "missing teeth" OR "caries" OR "carious").ab,ti,kf.	290,494

Web of Science (Core Collection) Search Results – 08 Jan 2026

Search	Query	Results
#5	#1 AND (#2 OR #3 OR #4)	1,019
#4	TS=(((("disab*" OR "special need*") NEAR/3 ("person*" OR "patient*" OR "individual*" OR "people" OR "dental care")) OR "special care dentistry") NEAR/5 ("hearing" OR "speech" OR	1,944

Search	Query	Results
	"deaf" OR "auditory" OR "visual" OR "blind" OR "sensory" OR "special school*"))	
#3	TS=((("visual*" OR "vision*" OR "sensory") NEAR/3 ("impair*" OR "disorder*" OR "disab*" OR "loss*" OR "defect*" OR "damage" OR "handicap*" OR "function*" OR "dysfunction*")) OR "impaired vis*" OR "blindness*" OR "low vision*" OR "reduced vision*" OR "subnormal vision*" OR "abnormal vision*" OR "diminished vision*" OR "blind indiv*" OR "blind people" OR "blind person*" OR "blind worker*" OR "the blind" OR "loss of sight" OR "sighted" OR "poor sight" OR "low sight" OR "poor vision*" OR "reduced sight*")	235,002
#2	TS=((("hearing" OR "speech" OR "sensory") NEAR/3 ("impair*" OR "disorder*" OR "disab*" OR "loss*" OR "defect*" OR "damage" OR "handicap*" OR "function*" OR "dysfunction*")) OR ("hard-of-hearing" OR "deaf*" OR "auditory rehab*" OR "auditory disab*" OR "auditory impair*" OR "auditory handicap*" OR "hypoacus*" OR "hearing aid*" OR "hearing implant*" OR "hearing prosthes*" OR "hearing device*" OR "cochlear implant*" OR "cochlear prosthes*" OR "cochlear device*" OR ("auditory" AND ("implant*" OR "prosthes*")) OR "poor hearing" OR "reduced hearing" OR "subnormal hearing" OR "abnormal hearing" OR "diminished hearing"))	201,312
#1	TS=((("oral" OR "dental" OR "gingiv*" OR "gum*" OR "periodont*") NEAR/3 ("hygien*" OR "health" OR "status" OR "care")) OR "tooth loss" OR "edentul*" OR "missing teeth" OR "caries" OR "carious")	152,485
